# Supplementary material for: Extravascular optical coherence tomography of cerebral vessel walls in vivo
Source: PLoS One. 2023 May 17;18(5):e0276307. doi: 10.1371/journal.pone.0276307 (PMC10191299; doi:10.1371/journal.pone.0276307)
Supplement: Attachment — Submitted filename: Response to the Reviewers.docx [file pone.0276307.s001.docx]

# Comments according Journal Requirements

**1. Please ensure that your manuscript meets PLOS ONE's style requirements, including those for file naming. The PLOS ONE style templates can be found at**

[**https://journals.plos.org/plosone/s/file?id=wjVg/PLOSOne_formatting_sample_main_body.pdf**](https://journals.plos.org/plosone/s/file?id=wjVg/PLOSOne_formatting_sample_main_body.pdf)

Response: We adapted the formatting standards as set by the given “PLOSOne formatting sample”. This included size of level 1 headings (18pt) and level 2 headings (16pt). See example of adapted heading here:

# *Materials and Methods*

## ***Patients***

*10 patients (6 = female; 4 = male; mean age 54±19; range 17-77) with indication for supra tentorial, micro neurosurgery were included. A cerebral vasospasm (n = 1), superficial sylvian veins (n = 4) and cerebral arteries (n = 13) were selected for OCT scanning. Indication for craniotomy ranged from brain tumors (n = 4) to cerebral aneurysms (n = 6). All patients gave written informed consent. The study was approved by the local ethics committee (No. 3012-2016). Patient characteristics are listed in* ***Table 1****.*

Response: We adapted the formatting standards further by exclusion of “funding or competing interests’ information”. See example of new excluded “funding or competing interests’ information” here:

# Declarations

**Funding** The authors received no specific funding for this work.

**Conflicts of Interest** OptoMedical Technologies GmbH, Maria-Goeppert-Straße 9, 23562 Lübeck, Schleswig Holstein, Deutschland supported the study with free equipment for iOCT. This does not alter the adherence to PLOS ONE policies on sharing data and materials. The authors declare no employment, remunerated consultancy, involvements in patents, products in development or marketed products linked to this company. The authors have nothing further to disclose and no further competing interests.

**Ethics Approval** All procedures performed in this study involving human participants were in accordance with ethical standards of the institutional and/or national research committee and with the 1964 Helsinki declaration and its later amendments or comparable ethical standards. The study was further approved by the local ethics committee (No. 3012-2016).

**Informed Consent** Informed consent was obtained from all individual participants included in the study.

**Consent for Publication** Consent for publication was obtained from all individual participants included in the study.

**Authors’ Contribution Karl Hartmann:** Conceptualization, Methodology, Validation, Formal Analysis, Data Curation, Sketching, Writing, Editing, **Belal Neyazi**: Conceptualization, Investigation **Claudia A. Dumitru:** Reviewing and Editing **Aiden Haghikia:** Reviewing and Editing **I. Erol Sandalcioglu**: Conceptualization, Validation, Reviewing and Editing, Supervision **Klaus-Peter Stein**: Conceptualization, Validation, Visualization, Writing, Editing

**1. Please ensure that your manuscript meets PLOS ONE's style requirements, including those for file naming. The PLOS ONE style templates can be found at**

[**https://journals.plos.org/plosone/s/file?id=ba62/PLOSOne_formatting_sample_title_authors_affiliations.pdf**](https://journals.plos.org/plosone/s/file?id=ba62/PLOSOne_formatting_sample_title_authors_affiliations.pdf)

Response: The formatting style of the authors and affiliations were adapted according PLOSOne standards. Please note at this point that the order of the authors was adopted to better fit the contributions of each. This is in consensus with all authors. Detailed information is given in the “Authors´Contribution”section:

*Karl Hartmann^1^*, Belal Neyazi^1^, Claudia A. Dumitru^1^, Aiden Haghikia^2^, I. Erol Sandalcioglu^1^ and Klaus-Peter Stein^1^*

*^1^ Universitätsklinik für Neurochirurgie, Otto-von-Guericke-Universität Magdeburg, Sachsen-Anhalt, Magdeburg, Deutschland*

*^2^ Universitätsklinik für Neurologie, Otto-von-Guericke-Universität Magdeburg, Sachsen-Anhalt, Magdeburg, Deutschland*

*corresponding author: karl.hartmann@med.ovgu.de // ORCID:* ***0000-0001-6732-952X***

**Authors’ Contribution Karl Hartmann:** Conceptualization, Methodology, Validation, Formal Analysis, Data Curation, Sketching, Writing, Editing, **Belal Neyazi**: Conceptualization, Investigation **Claudia A. Dumitru:** Reviewing and Editing **Aiden Haghikia:** Reviewing and Editing **I. Erol Sandalcioglu**: Conceptualization, Validation, Reviewing and Editing, Supervision **Klaus-Peter Stein**: Conceptualization, Validation, Visualization, Writing, Editing

**2. Thank you for stating the following financial disclosure: "No funding was received."**

**At this time, please address the following queries:**

**a) Please clarify the sources of funding (financial or material support) for your study. List the grants or organizations that supported your study, including funding received from your institution.**

**b) State what role the funders took in the study. If the funders had no role in your study, please state: “The funders had no role in study design, data collection and analysis, decision to publish, or preparation of the manuscript.”**

**c) If any authors received a salary from any of your funders, please state which authors and which funders.**

**d) If you did not receive any funding for this study, please state: “The authors received no specific funding for this work.”**

**Please include your amended statements within your cover letter; we will change the online submission form on your behalf.**

Response: The predefined fraise was used:

# *Declarations*

***Funding*** *The authors received no specific funding for this work.*

**3. Thank you for stating the following in the Competing Interests section: "The authors have nothing to disclose and no competing interests. OptoMedical Technologies GmbH supported the study with free equipment for iOCT."**

**We note that you received funding from a commercial source: [Name of Company]**

**Please provide an amended Competing Interests Statement that explicitly states this commercial funder, along with any other relevant declarations relating to employment, consultancy, patents, products in development, marketed products, etc.**

**Within this Competing Interests Statement, please confirm that this does not alter your adherence to all PLOS ONE policies on sharing data and materials by including the following statement: "This does not alter our adherence to PLOS ONE policies on sharing data and materials.” (as detailed online in our guide for authors http://journals.plos.org/plosone/s/competing-interests). If there are restrictions on sharing of data and/or materials, please state these. Please note that we cannot proceed with consideration of your article until this information has been declared.**

**Please include your amended Competing Interests Statement within your cover letter. We will change the online submission form on your behalf.**

Response: We appreciate this detailed description of conflicts of interests. We now specified our statement:

***Conflicts of Interest*** *OptoMedical Technologies GmbH, Maria-Goeppert-Straße 9, 23562 Lübeck, Schleswig Holstein, Deutschland supported the study with free equipment for iOCT. This does not alter the adherence to PLOS ONE policies on sharing data and materials. The authors declare no employment, remunerated consultancy, involvements in patents, products in development or marketed products linked to this company. The authors have nothing further to disclose and no further competing interests.*

**4. In your Data Availability statement, you have not specified where the minimal data set underlying the results described in your manuscript can be found. PLOS defines a study's minimal data set as the underlying data used to reach the conclusions drawn in the manuscript and any additional data required to replicate the reported study findings in their entirety. All PLOS journals require that the minimal data set be made fully available. For more information about our data policy, please see http://journals.plos.org/plosone/s/data-availability.**

**Upon re-submitting your revised manuscript, please upload your study’s minimal underlying data set as either Supporting Information files or to a stable, public repository and include the relevant URLs, DOIs, or accession numbers within your revised cover letter. For a list of acceptable repositories, please see http://journals.plos.org/plosone/s/data-availability#loc-recommended-repositories. Any potentially identifying patient information must be fully anonymized.**

**Important: If there are ethical or legal restrictions to sharing your data publicly, please explain these restrictions in detail. Please see our guidelines for more information on what we consider unacceptable restrictions to publicly sharing data: http://journals.plos.org/plosone/s/data-availability#loc-unacceptable-data-access-restrictions. Note that it is not acceptable for the authors to be the sole named individuals responsible for ensuring data access.**

Response: We highly appreciate PLOSONE standards for reproducibility of results and open data sharing. We therefore made exemplary raw 3-D OCT volume scans of a human cerebral internal carotid artery and superficial silvian vein as well as an excel sheet demonstrating raw measurements of vessel wall layers freely available. The manuscript was adapted at several points:

*… of cerebral vessel wall modifications in 10 exemplary OCT B-scans of 10 different cerebral arteries and patients are shown in* ***Fig 2, Supp Inf****.*

*… vessel wall layer diameters are listed in* ***Table 2, Supp Inf****.*

*… mono layered composition in OCT scan,* ***5*** *intraluminal scattering of light,* ***6*** *trabecular system. See* ***Supp Inf*** *for raw 3-D OCT scan.]*

# *Supporting Information*

*A data set which demonstrates exemplary raw OCT volume scans of the first in vivo scans of a human cerebral internal carotid artery and superficial silvian vein vessel wall characteristics as well as an excel sheet with raw results of vessel wall layer diameters were made freely available to improve reproducibility of the results:*

[*https://osf.io/a3gx8/?view_only=649952d8965543349ffc3c90cf8d6aea*](https://osf.io/a3gx8/?view_only=649952d8965543349ffc3c90cf8d6aea)

**5. Please amend the manuscript submission data (via Edit Submission) to include author I. Erol Sandalcioglu.**

Response: Done

**6. Your ethics statement should only appear in the Methods section of your manuscript. If your ethics statement is written in any section besides the Methods, please delete it from any other section.**

Response: Done. Declarations section was excluded from the main manuscript:

# *Declarations*

***Funding*** *The authors received no specific funding for this work.*

***Conflicts of Interest*** *OptoMedical Technologies GmbH, Maria-Goeppert-Straße 9, 23562 Lübeck, Schleswig Holstein, Deutschland supported the study with free equipment for iOCT. This does not alter the adherence to PLOS ONE policies on sharing data and materials. The authors declare no employment, remunerated consultancy, involvements in patents, products in development or marketed products linked to this company. The authors have nothing further to disclose and no further competing interests.*

***Ethics Approval*** *All procedures performed in this study involving human participants were in accordance with ethical standards of the institutional and/or national research committee and with the 1964 Helsinki declaration and its later amendments or comparable ethical standards. The study was further approved by the local ethics committee (No. 3012-2016).*

***Informed Consent*** *Informed consent was obtained from all individual participants included in the study.*

***Consent for Publication*** *Consent for publication was obtained from all individual participants included in the study.*

***Authors’ Contribution Karl Hartmann:*** *Conceptualization, Methodology, Validation, Formal Analysis, Data Curation, Sketching,* *Writing, Editing,* ***Belal Neyazi****: Conceptualization, Investigation* ***Claudia A. Dumitru:*** *Reviewing and Editing* ***Aiden Haghikia:*** *Reviewing and Editing* ***I. Erol Sandalcioglu****: Conceptualization, Validation, Reviewing and Editing, Supervision* ***Klaus-Peter Stein****: Conceptualization, Validation, Visualization, Writing, Editing*

# Comments to the 1^st^ Reviewer

**Reviewer #1: In the present manuscript, Hartman et al apply in vivo extravascular microscope integrated OCT (iOCT) of the cerebral vessel wall during neurosurgical procedures to analyze cerebral vessel wall layers of both arteries and veins. Supported by illustrative figures, they show that iOCT is a feasible technique. They furthermore provide measures of different cerebral vessel wall layers in vivo for the first time. The topic is of relevance for the field and is both interesting and relevant for neurologists and neurosurgeons since the technique of iOCT might allow to address open pathophysiological questions of cerebral vessel diseases. The methods are described in detail and are sound. The results are presented adequately and support the derived conclusions.**

Response: We appreciate the positive evaluation of the manuscript.

**I have one major and some minor comments which I hope the authors can address in a revise version of the manuscript.**

**MAJOR:**

**Results: Please describe whether any complications or safety issues occurred during iOCT analysis. Since iOCT is a medical procedure involving humans, addressing its safety and risk profile is crucial.**

Response: OCT imaging depends on backscattered infrared light. Its electromagnetic energy is lower than the one of visual light. Therefore, its capability for electronic excitation of molecules also remains lower. Leading to lesser tissue irritation then visual light. We now clarified this in the method section:

*OCT imaging depends on near infrared light resulting in less electronic excitation then the visible light spectrum and is therefore harmless to biological tissue^5^. In biological tissue penetrating depth is approx. 4000 μm and therefore suitable for cerebral vessel wall imaging^7^.*

**MINOR:**

**Methods: To allow reproducibility of the findings, the authors should describe in detail where they exactly analyzed the respective vessels. For example, regarding MCA/ACA/ICA, the authors may provide which segments have been measured by iOCT. This information might be included into table 1.**

Response: We appreciate this suggestion which can improve the accuracy of the manuscript. Table 1 and the Method section has been amended accordingly:

*PLACE [Table 1. Patient Characteristics]*

| ***No.*** | ***Age*** | ***Sex*** | ***Scanned Vessel*** | ***Scanned Segment*** | ***Indication for Surgery*** |
| --- | --- | --- | --- | --- | --- |
| *1* | *66* | *f* | *ICA* | *C1* | *Tumor extra-axial* |
| *2* | *77* | *m* | *MCA* | *M2* | *Tumor extra-axial* |
| *3* | *71* | *m* | *ICA*  *SV* | *C1* | *Tumor extra-axial* |
| *4* | *17* | *f* | *ICA* | *C1* | *Tumor intra-axial* |
| *5* | *75* | *m* | *ACA*  *ICA* | *A1*  *C1* | *Cerebral Aneurysm*  *AcomA* |
| *6* | *46* | *f* | *ACA* | *A1* | *Cerebral Aneurysm*  *ACA* |
| *7* | *58* | *f* | *ICA*  *SV* | *C2* | *Cerebral Aneurysm*  *ICA* |
| *8* | *44* | *f* | *ICA*  *MCA* | *C1*  *M2* | *Cerebral Aneurysms*  *ICA, MCA bilateral* |
| *9* | *56* | *f* | *ICA*  *SV* | *C1* | *Cerebral Aneurysms*  *ICA, MCA* |
| *10* | *29* | *m* | *ICA*  *ICA*  *SV* | *C1*  *C2* | *Cerebral Aneurysms*  *ICA* |

*LEGEND TABLE 1[Displaying patients’ characteristics with definition of age, gender, scanned vessels and indications for neurosurgical interventions. Note that definition of scanned segments was limited by the surgical approach but it was aimed for proximal segments of the anterior Circle of Willis. Abbreviations: Internal carotid artery (ICA), middle cerebral artery (MCA), anterior cerebral artery (ACA), anterior communicating artery (AcomA) and sylvian vein (SV).]*

*... The OCT scan site was defined by a senior vascular neurosurgeon with experience in OCT scanning. Focusing on the proximal branches of the anterior Circle of Willis with limitation due to the neurosurgical approach. An orthograde scan-angle and highest microscope magnification ...*

**Results: In Table 1, the authors used the abbreviations “ICA” and “ACI”, whereas “ACI” is not defined within the table legend. I suggest that ACI might also reflect ICA, please clarify and revise accordingly. Please use the same abbreviations for the same vessel structures in all provided tables and figures (table 1: ICA, table 2: ACI).**

Response: Both tables were revised as suggested by the reviewer:

*PLACE [Table 1. Patient Characteristics]*

| ***No.*** | ***Age*** | ***Sex*** | ***Scanned Vessel*** | ***Scanned Segment*** | ***Indication for Surgery*** |
| --- | --- | --- | --- | --- | --- |
| *1* | *66* | *f* | *ICA* | *C1* | *Tumor extra-axial* |
| *2* | *77* | *m* | *MCA* | *M2* | *Tumor extra-axial* |
| *3* | *71* | *m* | *ICA*  *SV* | *C1* | *Tumor extra-axial* |
| *4* | *17* | *f* | *ICA* | *C1* | *Tumor intra-axial* |
| *5* | *75* | *m* | *ACA*  *ICA* | *A1*  *C1* | *Cerebral Aneurysm*  *AcomA* |
| *6* | *46* | *f* | *ACA* | *A1* | *Cerebral Aneurysm*  *ACA* |
| *7* | *58* | *f* | *ICA*  *SV* | *C2* | *Cerebral Aneurysm*  *ICA* |
| *8* | *44* | *f* | *ICA*  *MCA* | *C1*  *M2* | *Cerebral Aneurysms*  *ICA, MCA bilateral* |
| *9* | *56* | *f* | *ICA*  *SV* | *C1* | *Cerebral Aneurysms*  *ICA, MCA* |
| *10* | *29* | *m* | *ICA*  *ICA*  *SV* | *C1*  *C2* | *Cerebral Aneurysms*  *ICA* |

*LEGEND TABLE 1[Displaying patients’ characteristics with definition of age, gender, scanned vessels and indications for neurosurgical interventions. Note that definition of scanned segments was limited by the surgical approach but it was aimed for proximal segments of the anterior Circle of Willis. Abbreviations: Internal carotid artery (ICA), middle cerebral artery (MCA), anterior cerebral artery (ACA), anterior communicating artery (AcomA) and sylvian vein (SV).]*

*PLACE [Table 2. Vessel Wall Diameters and Proportions of Cerebral Arteries]*

| ***No.*** | ***Artery*** | ***Vessel Wall Diameter***  *absolute in µm* | ***Tunica***  *in µm (% of absolute vessel wall diameter)* | | | | | |
| --- | --- | --- | --- | --- | --- | --- | --- | --- |
|  |  |  | ***Intima*** | | ***Media*** | | ***Externa*** | |
| *1* | *ICA* | *60* | *20* | *(33)* | *20* | *(34)* | *20* | *(33)* |
| *2* | *ICA* | *400* | *120* | *(30)* | *130* | *(33)* | *150* | *(37)* |
| *3* | *ICA* | *210* | *50* | *(24)* | *60* | *(28)* | *100* | *(48)* |
| *4* | *ICA*  *ICA* | *300*  *450* | *80*  *130* | *(27)*  *(29)* | *180*  *220* | *(60)*  *(49)* | *40*  *100* | *(13)*  *(22)* |
| *5* | *ICA* | *310* | *60* | *(20)* | *140* | *(45)* | *110* | *(35)* |
| *6* | *ACA*  *ICA* | *370*  *340* | *160*  *90* | *(43)*  *(26)* | *140*  *190* | *(38)*  *(56)* | *70*  *60* | *(19)*  *(18)* |
| *7* | *ACA* | *280* | *110* | *(39)* | *140* | *(50)* | *30* | *(11)* |
| *8* | *ICA*  *ACI* | *120*  *320* | *40*  *50* | *(33)*  *(16)* | *50*  *140* | *(42)*  *(44)* | *30*  *130* | *(25)*  *(40)* |
| *9* | *ICA* | *520* | *100* | *(19)* | *290* | *(56)* | *130* | *(25)* |
| *10* | *MCA* | *170* | *80* | *(47)* | *50* | *(29)* | *40* | *(24)* |
|  | ***MEAN*** | *296±125* | *84±38* | *30±9* | *134±73* | *43±10* | *78±43* | *27±11* |
|  | ***RANGE*** | *60-520* | *20-160* | *16-47* | *20-290* | *28-60* | *20-150* | *11-48* |

*LEGEND TABLE 2[Absolute vessel wall diameters of 10 cerebral arteries on the right. Notice the high range of inter-individual variability. Absolute and relative diameters of cerebral vessel wall layers on the right. Mean values and ranges are listed in the bottom rows. Notice the pronounced diameter of tunica media. Notice the wide span in range in all three vessel wall layers reflecting the inter-individual variability. See* ***Supp Inf*** *for raw data.]*

**Results: I have doubts whether it is useful to provide a mean vessel diameter in table 2 which is derived from three different anatomical vessel structures (ACI, ACA, MCA). I suggest providing mean vessel diameters of specific cerebral vessels that were analyzed in comparable segments and regions (see also above).**

Response: We see the point of the reviewer and appreciate the suggestion. But detailed analysis of our primary data showed no consistency or trends of vessel wall diameters in relation to anatomical parts of the cerebral vasculature e. g. C1 segment of ICA (see Supp. Inf. Excel sheet). Therefore - and because of the limited number of measured vessel walls in general - we decided to display the absolute results in Table 2.

**Results: Did the authors recognize any associations of their iOCT findings with age and sex of the respective patients?**

Response: The limited number of participants inhibits a statistical significant analysis of such correlations. Therefore, we would like to exclude such statements from the manuscript. But also descriptively during data analysis such tendencies were not observed.

**Discussion: The authors claim to provide the first in vivo measures of cerebral vessel layers derived from OCT-A. Please discuss whether the findings from iOCT correspond to histological studies of comparable vessels and comparable regions.**

Response: We thank the reviewer for this suggestion to improve our manuscript. We now provide more details on the correlation between histological studies and OCT in the “Introduction” and “Discussion” sections, respectively:

*Imaging of cerebral vessel wall characteristics is fundamental to understand pathophysiological processes of cerebrovascular diseases and for intraoperative guidance during micro neurosurgery. Histology - albeit its’ superior and unexcelled resolution - excludes an in vivo approach and is further constrained by numerous process-related artifacts, such as specimen shrinkage, tissue damage and tissue separation^1^*

*It shows an exceedingly high spatial resolution ranging from 1 - 15 µm and therefore allows for identification of structural constituents to the extent of histology^7^. In ex vivo experimental set ups the correlation of intravascular OCT and histological findings of vessel walls could already be demonstrated ^8^ ^9^ ^10^. In this context, extravascular OCT even seemed to exceed intravascular OCT in terms of image quality ^11^ ^12^ ^13^. Physically depending on light, microscope integration is fairly simple…*

*exceptional high spatial resolution - in our set up 7.5 µm - and penetration depth ranges from 1000 – 3000 µm^16^. In line with this resolution experimental OCT analysis of vessel walls could priorly demonstrate coherence with histological findings^8^ ^9^ ^10^. ...*

**Discussion: Future perspectives: From my point of view, there are several more fields that could be addressed by iOCT. For example, pathophysiological features and findings in cerebral aneurysms (especially after successful clipping) would be very favorable.**

Response: With this manuscript we would like to focus primarily on the possibilities of the technique according fundamental research on dynamic microanatomy in vivo and pathological alterations of such like arteriosclerosis.

But we also see the value of the technique in several domains of microvascular surgery like aneurysm, bypass, arteriovenous malformation as well as cranial and spinal arteriovenous fistula surgeries. We therefore expanded the “Future Perspectives” section of the manuscript:

*Microscope-integrated extravascular OCT should be further valued as an intraoperative imaging tool in the context of cardiac and neurosurgery. The highly demanding field of vascular neurosurgery with pathologies ranging from cerebral aneurysms, cerebral as well as spinal arterio-venous-malformations and Moyamoya disease could be targeted among others. In the latter, OCT could explicitly delineate the optimal site for vascular harvest and extra-intra cranial bypass ^34^.*

# Comments to the 2^nd^ Reviewer

**Reviewer #2: The authors use of microscope integrated optical coherence tomography (OCT) is similar to the groups prior published papers. Their prior publications show that OCT can be successfully used in-vivo to demonstrate baseline characteristics of a particular pathology such as an aneurysm or an arachnoid cyst. Outside of the novel use of this application, which they have already demonstrated, it is difficult to ascertain what they are trying to show with their data.**

Response: The results presented in this study are the first extravascular OCT scans and measurements of cerebral vessel wall layers in vivo – and for this reason alone they need to be reported. The application of OCT in this domain – in comparison to other neurosurgical domains – is unique since it demonstrates the (a) value of OCT as a fundamental research tool for microanatomy and clinical highly relevant pathologies of vessel walls e. g. arteriosclerosis, (b) the variability of vessel wall layer diameters among individuals, (c) the variability of arteriosclerotic alterations of cerebral vessel walls, (d) the variation of endo-luminal diameters and diameters of single cerebral vessel wall layers in cerebral vasospasm and (e) the possibility of visualizing turbulent blood flow. These five points are demonstrated in the manuscript.

**There is already limited information in the presented patient characteristics, and no attempt to demonstrate consistency within the presented factors. Even the comparison of vessel wall and vessel wall layers do not all compare the same type of arteries,**

Response: A detailed range of patient characteristics were analyzed. But relevant tendencies were not observed and therefore not reported. Furthermore, the number of participants itself inhibits relevant statistical analysis.

Naturally, only vessels which were exposed to the individual surgical approach could be scanned, leading to a certain degree of variability. But the surgeons focused on proximal branches of the anterior Circle of Willis to ensure comparability. This is now explained in more detail in the manuscript, see below.

We also appreciate the remark about vessel segment specification. Therefore, Table 1 and its header were edited to demonstrate the scanned segments. This highlights that mainly the C1 Segment of ICA and near proximal branches were scanned outlining the comparability of vessel wall diameter measurements:

*PLACE [Table 1. Patient Characteristics]*

| ***No.*** | ***Age*** | ***Sex*** | ***Scanned Vessel*** | ***Scanned Segment*** | ***Indication for Surgery*** |
| --- | --- | --- | --- | --- | --- |
| *1* | *66* | *f* | *ICA* | *C1* | *Tumor extra-axial* |
| *2* | *77* | *m* | *MCA* | *M2* | *Tumor extra-axial* |
| *3* | *71* | *m* | *ICA*  *SV* | *C1* | *Tumor extra-axial* |
| *4* | *17* | *f* | *ICA* | *C1* | *Tumor intra-axial* |
| *5* | *75* | *m* | *ACA*  *ICA* | *A1*  *C1* | *Cerebral Aneurysm*  *AcomA* |
| *6* | *46* | *f* | *ACA* | *A1* | *Cerebral Aneurysm*  *ACA* |
| *7* | *58* | *f* | *ICA*  *SV* | *C2* | *Cerebral Aneurysm*  *ICA* |
| *8* | *44* | *f* | *ICA*  *MCA* | *C1*  *M2* | *Cerebral Aneurysms*  *ICA, MCA bilateral* |
| *9* | *56* | *f* | *ICA*  *SV* | *C1* | *Cerebral Aneurysms*  *ICA, MCA* |
| *10* | *29* | *m* | *ICA*  *ICA*  *SV* | *C1*  *C2* | *Cerebral Aneurysms*  *ICA* |

*LEGEND TABLE 1[Displaying patients’ characteristics with definition of age, gender, scanned vessels and indications for neurosurgical interventions. Note that definition of scanned segments was limited by the surgical approach but it was aimed for proximal segments of the anterior Circle of Willis. Abbreviations: Internal carotid artery (ICA), middle cerebral artery (MCA), anterior cerebral artery (ACA), anterior communicating artery (AcomA) and sylvian vein (SV).]*

*... The OCT scan site was defined by a senior vascular neurosurgeon with experience in OCT scanning. Focusing on the proximal branches of the anterior Circle of Willis with limitation due to the neurosurgical approach. An orthograde scan-angle and highest microscope magnification was intended...*

**and their only reasonable conclusion is that artery sizes vary.**

Response: In the discussion we pointed out physiological, pathophysiological as well as methodological factors which might influence the variability of vessel wall and vessel wall layer diameters. We now edited this paragraph for more clearness:

*To the best of our knowledge, we here present the first in vivo measurements of cerebral vessel wall and single layer diameters. Absolute as well as proportional measurements varied widely. Physiological, pathological and methodological factors might influence these measurements. Physiologically we are looking at individual variants of vascular size as well as individual vessel wall layer diameters. Furthermore, we are scanning different vascular tones. Vascular tone not only influences the absolute diameter of vessels but also the diameter of its three tunicae and the proportions among them. This physiological mechanism was remarkably illustrated by scanning of an incidental intraoperative vasospasm, see* ***Fig 4****. Pathological factors like arteriosclerosis lead to an increased diameter of tunica intima and in late stages to a diminished diameter of tunica media due to muscular atrophy influencing the vessel wall and vessel wall layer diameters ^29 30^. This variability of pathological alterations is well demonstrated in* ***Fig 2****. Methodological factors could be slight deviation from an orthograde scan angel due to the limited surgical approach and - of course - the limited case number.*

**Overall, the authors show this particular use of OCT is possible, which has already been demonstrated, but fail to bring any new information regarding this technique.**

Response: The applicability of microscope integrated OCT in neurosurgical and specific vascular neurosurgical settings was demonstrated elsewhere in detail (Böhringer 2009, Lankenau 2013, Hartmann 2019). This manuscript focuses instead on the validation of this imaging technique for cerebral vessel walls and on the discussion of these results.
